# Outcomes of Pregnancy Termination of Dead Fetus in Utero in Second Trimester by Misoprostol with Various Regimens

**DOI:** 10.3390/ijerph191912655

**Published:** 2022-10-03

**Authors:** Saipin Pongsatha, Nuchanart Suntornlimsiri, Theera Tongsong

**Affiliations:** Department of Obstetrics and Gynecology, Faculty of Medicine, Chiang Mai University, Chiang Mai 50200, Thailand

**Keywords:** dead fetus in utero, pregnancy termination, second trimester, misoprostol

## Abstract

*Objective:* To determine the efficacy and adverse outcomes of misoprostol with various regimens for the second-trimester-pregnancy termination of a dead fetus in utero (DFIU). *Patients and Methods:* A retrospective descriptive study, based on the prospective database, was conducted on pregnancies with dead fetuses in utero in the second trimester. All patients underwent pregnancy termination with various regimens of misoprostol. *Results:* A total of 199 pregnancies meeting the inclusion criteria were included. The mean age of the participants and the mean gestational age were 30.2 years and 21.1 weeks, respectively. The two most common regimens were 400 mcg injected intravaginally every six hours and 400 mcg taken orally every four hours. In the analysis of the overall efficacy, including all regimens, the mean fetal delivery time was 18.9 h. When considering only the cases involving a delivery within 48 h (success cases), the mean fetal delivery time was 13.6 h. The rates of fetal delivery for all cases at 12, 24, 36, and 48 h were 50.3%, 83.8%, 89.3%, and 93.9%. In the comparison between the various regimens, there were no significant differences in the rate of fetal delivery at 12, 24, 36, and 48 h and adverse effects such as chill, diarrhea, nausea, vomiting, and other parameters such as the requirement for intravenous analgesia, the requirement for curettage for incomplete abortions, the mean total dose of misoprostol, and the rate of postpartum hemorrhage (PPH). Nevertheless, the rate of fever was significantly higher in the regimen of intravaginal insertion of 400 mcg every six hours and that of the requirement for oxytocin was significantly higher in the regimen of oral supplementation of 400 mcg every four hours. *Conclusions:* The overall success rate within 48 h was 93.6%, which was not different among the various misoprostol regimens. In addition, there were no significant differences in the mean fetal delivery times and the rates of fetal delivery at 12, 24, 36, and 48 h. However, some parameters such as fever, oxytocin requirement, and mean total dose of misoprostol were statistically significant between regimens. In the aspect of global health, misoprostol can be a good option in clinical practice, especially in geographical areas with low-resource levels.

## 1. Introduction

A dead fetus in utero (DFIU) is an unexpected event and is stressful for all pregnant women. Sometimes the cause of death can be found but other times it cannot. However, in any situation after fetal death, the termination of pregnancy should not be delayed more than two to three weeks after the diagnosis to prevent maternal consumptive coagulopathy. More importantly, a delayed termination can greatly increase the stress in women or couples due to carrying a dead fetus in utero or the nature of a hopeless pregnancy, leading to deteriorating mental health. The rate of stillbirth in the second and third trimesters in Taiwan was 0.98%, of which 55.4% of stillbirths occurred in the second trimester while 44.6% occurred in the third trimester [1]. Whereas the rate of stillbirth in the United Kingdom was 0.4% [2]. The exact rate of second-trimester DFIU in our country (Thailand) is not known. Nevertheless, it seems to be relatively rare from our observations. The termination of pregnancy (TOP) without delay is challenging, especially in low-resource countries where surgical procedures, blood component products, or operation rooms are not always available. Therefore, the techniques for the termination of a DFIU with a low cost, high safety, and high effectiveness are yet to be determined, especially in cases of an unfavorable cervix, which is the key point for success. The methods for the TOP in the second trimester can be performed by mechanical, surgical, and medical methods. The most preferred method is the medical method, as it is less invasive and more natural than the others. The combination of mifepristone followed by intravaginal misoprostol shows a better outcome than misoprostol alone [3,4]. However, with the limitation of mifepristone accessibility in many countries, misoprostol alone is still a well-known and promising drug for the termination of pregnancy both in live fetuses [5] and DFIU [6,7,8]. Until now, though several studies of misoprostol on the TOP have been published, there has been limited data concerning the most effective dosage and the best route of administration for the second-trimester termination of a DFIU. While the intravaginal route is the most popular method, it possesses a conflicting effectiveness when compared with the oral route [9]. We have developed a prospective database of TOP with misoprostol since 1997, either in cases of live or dead fetuses. Thus, we had a great opportunity to take advantage of the database to perform this comparative study, which aimed to determine the efficacy and complications of the TOP using various regimens of misoprostol.

## 2. Patients and Methods

This is a retrospective comparative study based on our prospective database to compare the effectiveness of various misoprostol regimens in termination of pregnancy with dead fetuses in the second trimester, conducted at the Department of Obstetrics and Gynecology, Faculty of Medicine, Chiang Mai University, Thailand, from 2003 to 2021. The prospective database was developed in 1997 to collect all consecutive cases of misoprostol use in our department and the participants provided written informed consent. The present study was conducted with ethical approval by the Institutional Review Boards, Faculty of Medicine, Chiang Mai University (Research Study ID 8894). The pregnant women recruited into the study met the following criteria: (1) diagnosis of a second trimester DFIU, confirmed by ultrasound examination; (2) gestational age between 14 and 28 weeks of pregnancy; (3) unfavorable cervix, defined as Bishop scores of <5; and (4) the participants were not in labor (no regular contraction with cervical changes). The exclusion criteria were as follows: (1) twin pregnancy; (2) spontaneous labor (regular uterine contraction) occurring before misoprostol administration; (3) contraindication for vaginal delivery such as placenta previa totalis; (4) more than one previous cesarean section. However, the pregnant women with one previous cesarean delivery or myomectomy were not excluded from the study. All participants received misoprostol alone to enable cervical ripening (with various regimens based on different periods of data collection) for TOP. The regimens included intravaginal route and other routes with various dosages and intervals of drug administration, mostly based on the physicians’ preference.

Clinical and demographic data were prospectively collected and recorded, including baseline characteristics of participants (maternal age, parity, gestational age, cervical Bishop score, etc.), misoprostol regimens (400 mcg intravaginally every 6 h, 400 mcg orally every 4 h, 400 mcg intravaginally followed by 400 mcg orally every 4 h, and others), adverse effects related to misoprostol (fever, chills, and diarrhea), intravenous analgesia and oxytocin requirement, estimated blood loss, uterine rupture, postpartum hemorrhage and total dose of misoprostol used, mean fetal delivery time, and the rate of fetal delivery within 12, 24, 36, and 48 h after the initiation of misoprostol. A successful termination was defined as fetal delivery within 48 h. The demographic data were prospectively collected and recorded in the record research form during recruitment, whereas the clinical data of management from medical records were comprehensively reviewed by the research team and recorded shortly after the final outcomes were known.

**Statistical analysis:** Statistical procedures were performed using the statistical package for the social sciences (SPSS) software version 26.0 (IBM Corp. Released 2019. IBM SPSS Statistics for Windows, Armonk, NY: IBM Corp). The baseline data were presented as mean ± SD or median (IQR) for continuous data, and as percentage for categorical data. In the comparisons, Chi-square tests were used for the categorical data, whereas ANOVA test was used for the continuous data. A *p*-value of less than 0.05 was defined as statistical significance.

## 3. Results

A total of 199 participants with a second trimester DFIU were recruited. The baseline characteristics in terms of maternal age, gestational age, Bishop scores, previous major uterine surgery, and details of the misoprostol regimen are presented in Table 1. The two most common regimens were 400 mcg administered intravaginally every six hours and 400 mcg orally every four hours. The rates of the overall side effects of misoprostol and adverse outcomes are presented in Table 2. The most common side effects were chills (37.9%), followed by fever (32.3%) and diarrhea (22.2%). It is noteworthy that TOP with misoprostol necessitated intravenous analgesia, oxytocin requirement (10 units of oxytocin in 1000 mL in normal saline start from 2 mU and not more than 20 mU/min by automatic infusion pump), and curettage in cases of incomplete termination after the delivery of the fetus in 31.8%, 11.6% and 19.2% of cases, respectively. Table 3 shows comparisons of the rates of adverse effects among various regimens. Whereas most adverse outcomes were comparable, the rate of fever was significantly higher in the 400 mcg-administered-intravaginally-every-4 h group (43.3%) and that of the oxytocin requirement was significantly higher in the 400 mcg-administered-orally-every-4 h group.

The accumulated rate of fetal delivery for all participants at 12, 24, 36, and 48 h was 50.3%, 83.8%, 89.3%, and 93.9%, respectively, as shown in Table 4. Approximately 50% of cases had successful fetal delivery within 12 h after the initiation of misoprostol and more than 83% were successful within 24 h. Successful cases (defined as fetal delivery within 48 h after misoprostol initiation) represented 93.9% of the patients.

Upon a comparison of the rates of fetal delivery within 12, 24, 36, and 48 h between the various regimens, no statistical significances were detected, as presented in Table 5. Of the successful cases (185; 92.9%), the rates of fetal delivery within 12, 24, 36, and 48 h were 54.1%, 89.2%, 95.1%, and 100% respectively, as presented in Table 6. Table 7 presents the mean fetal delivery time and the rate of failure to deliver within 48 h among the regimens. The regimen of 400 mcg administered intravaginally every 6 h seems to be better than other regimens in terms of a shorter mean fetal-delivery time and a lower rate of failure to deliver within 48 h, though such differences did not reach a statistical significance.

The mean fetal delivery time for all the participants and the successful cases was 18.9 h and 13.6 h, respectively, as shown in Table 8.

This study included six (3.0%) pregnancies involving a previous cesarean section. All of them had successful terminations within 48 h without uterine rupture or serious complications.

In this series, one case needed an emergency hysterectomy due to active vaginal bleeding after one dose of 400 mcg of misoprostol administered intravaginally at 25 weeks of gestation. This case was a primiparous woman possessing complications including a placenta previa marginalis and a large cervical leiomyoma (8 cm). Thus, the baseline nature of this case presented a very high risk for hemorrhage both during labor and postpartum and might not have been safe for the TOP, either with medical, mechanical, or surgical methods (evacuation). Due to the patient’s primiparous status, we wanted to try the medical method and hoped that it might be successful. Unfortunately, active bleeding in a difficult situation led to an emergency hysterectomy to save her life.

**Table 1:** Baseline Characteristics of Participants and Regimen of Misoprostol (199 Cases).

**Baseline Characteristics**	**Mean** **±** **SD**
Age (years)	30.2 ± 6.9
Gestational age (weeks)	21.1 ± 4.4
Initial Bishop score	1.7 ± 1.0
Nulliparous (N%)	111 (55.8%)
Previous cesarean section (N%)	6 (3.0%)
**Regimens of misoprostol**	**Number (%)**
400 vg q 6 h	67 (33.7)
400 oral q 4 h	66 (33.2)
400 vg then 400 oral q 4 h	37 (18.6)
400 vg q 4 h	15 (7.5)
Others	14 (7.0)

**Table 2:** Side Effects and adverse outcomes for All (198 Cases: Missing 1 case).

	Number (%)
Fever (BT > 38.0 C)	64 (32.3)
Chills	75 (37.9)
Diarrhea	44 (22.2)
Nausea	20 (10.1)
Vomiting	13 (6.6)
Oxytocin requirement	23 (11.6)
Requirement for intravenous analgesia	63 (31.8)
Curettage required for incomplete abortion	38 (19.2)
Blood loss > 500 mL	4 (2.0)
Uterine rupture	0
Mean total dose of misoprostol (mcg)	1191.8 ± 1136.9; (75–10,800)

**Table 3:** Adverse Effects between Regimens (198 Cases: Missing 1 case).

Adverse Effects N (%)	400 vg q 6 h	400 Oral q 4 h	400 vg, then 400 Oral q 4 h	Others	Chi-Square
Fever (BT > 38.0 °C)	29 (43.3%)	12 (18.2%)	14 (37.8%)	9 (32.1%)	0.017
Chills	22 (32.8%)	23 (34.9%)	19 (51.4%)	11 (39.3%)	0.277
Diarrhea	11 (16.4%)	19 (28.8%)	10 (27.0%)	4 (14.3%)	0.215
Nausea	3 (4.5%)	12 (18.2%)	3 (8.1%)	2 (7.1%)	0.057
Vomiting	3 (4.5%)	6 (9.1%)	2 (5.4%)	2 (7.1%)	0.739
Oxytocin requirement	3 (4.5%)	14 (21.2%)	4 (10.8%)	2 (7.1%)	0.020
Intravenous analgesia requirement	20 (29.9%)	21 (31.8%)	13 (35.1%)	9 (32.1%)	0.958
Curettage required for incomplete abortion	15 (22.4%)	9 (13.6%)	8 (21.6%)	6 (21.4%)	0.575
Blood loss > 500 mL	0	3 (4.5%)	1 (2.7%)	0	0.248
Uterine rupture	0	0	0	0	-
Mean total dose of misoprostol (mcg)	949.3 ± 742.9	1421.2 ± 1129.3	1524.3 ± 1707.1	806.0 ± 753.9	0.006 *

* ANOVA test.

**Table 4:** Fetal Delivery time for all (197 Cases: Missing 1 case; Hysterectomy 1 case).

Fetal Delivery at (h)	Number (%)
12 h	100 (50.3)
24 h	165 (83.8)
36 h	176 (89.3)
48 h	185 (93.9)

**Table 5:** Fetal delivery time between regimens (197 Cases: missing 1 case; hysterectomy 1 case).

Fetal Delivery at (h)	400 vg q 6 h N%	400 Oral q 4 h N%	400 vg then 400 Oral q 4 h N%	Others N%	Chi-Square
12 hr	33 (50)	33 (50)	19 (51.4)	15 (53.6)	0.989
24 hr	55 (83.3)	55 (83.3)	31 (83.8)	24 (85.7)	0.992
36 hr	58 (87.9)	59 (89.4)	33 (89.2)	26 (92.9)	0.916
48 hr	63 (95.5)	61 (92.4)	34 (91.9)	27 (96.4)	0.776

**Table 6:** Fetal delivery time for success cases (Fetal Del < 48 h = 185 cases).

Fetal Delivery at (h)	Number (%)
12 h	100 (54.1)
24 h	165 (89.2)
36 h	176 (95.1)
48 h	185 (100)

**Table 7:** Mean Fetal delivery time and rate of failure to deliver within 48 h (197 Cases: Missing 1 case; Hysterectomy 1 case).

Regimen	Mean Fetal Delivery Time (h)	Failure to Deliver within 48 h (Number %)
400 vg q 6 h	17.0 ± 17.1	3 (4.5)
400 oral q 4 h	20.6 ± 28.6	5 (7.6)
400 vg then 400 oral q 4 h	21.0 ± 31.3	3 (8.1)
Other regimens	14.1 ± 11.3	1 (3.6)

**Table 8:** Mean fetal and placental delivery time.

Parameters	Hours
Mean fetal delivery time for all (197 cases): missing 1 case, hysterectomy 1 case	18.9 ± 29.9
Mean fetal delivery time for successful case (185 cases) (fetal delivery within 48 h)	13.6 ± 9.1
Mean placental delivery time for all 197 cases: missing 1 case, hysterectomy 1 case	19.4 ± 24.7
Mean placental delivery time for successful cases 185 cases (fetal delivery within 48 h)	14.5 ± 11.1

## 4. Discussion

The TOP for second trimester DFIU with misoprostol was effective with a high safety profile. Though misoprostol combined with mifepristone is reported to have a higher efficacy than misoprostol alone for the TOP involving live fetuses [10,11], the studies on TOP with misoprostol in DFIU have been reported in very limited numbers [12]. Based on the finding that fetal delivery within 48 h occurred for 93.9% of all participants and that the mean fetal delivery time among the successful cases was 13.6 h, our findings indicate that misoprostol alone can also be highly effective and is promising for use as a primary method in the TOP of DFIU, especially in low-resource countries, because of its high efficacy, high safety, low cost, stability at room temperatures, and availability.

Our findings indicate that various regimens of misoprostol alone have comparable effectiveness in terms of success rate within 48 h and mean fetal delivery time, though the rate of side effects might be significantly different among regimens. The effectiveness in this study may not be applied to the TOP of viable fetuses because DFIU cases tend to have higher responses to misoprostol than those in live fetuses, as demonstrated in a previous study [13]. Our results are in agreement with the Cochrane Systematic Review [14], which states that the misoprostol alone regimen fails to show the difference in efficacy for the termination of DFIU pregnancies between the vaginal and sublingual or vaginal and oral routes. Nevertheless, the Cochrane review included only cases with fetal death before 24 weeks of gestation.

We did not directly compare the efficacy of misoprostol in the TOP of live fetuses with that of dead fetuses. Nevertheless, our previous study showed that the regimen of 400 mcg administered intravaginally every six hours in a live fetus had the mean abortion time of 19.9 h [15], which was slightly longer than that for the DFIU cases in this study (17 h). The findings suggested that with the same misoprostol regimen for the TOP in the second trimester, the DFIU probably provide a better response than live fetuses. It is possible that a DFIU itself tends to develop spontaneous uterine contraction, facilitating a more rapid delivery time or a higher rate of success than those of live fetuses.

The main finding of this study is that misoprostol alone is highly effective for the TOP of a DFIU with a high safety profile. Either route shows comparative efficacy, and 400 mcg may be the optimal dose for the TOP in the second trimester. Misoprostol either intravaginally, orally, or through a combined route shows a similar efficacy. No serious adverse effects or uterine ruptures were found, even though six participants in the present study had a previous uterine scar. However, strict precautions should be considered in every case, especially in cases with a previous uterine scar. This demonstration of safety cannot be supported by the small number of participants because normally the rate of uterine rupture is rare.

To date, the best regimen of misoprostol for second trimester terminations is not well-established. Based on extensive experience and the literature review, misoprostol at 400 mcg administered vaginally every 3–6 h is probably the optimal regimen. The larger dosage is likely to have more side effects, especially diarrhea, whereas the smaller dosage seems to be less effective. However, the oral route is also commonly practiced because of patients’ preference. Such reasons might partly explain why the two regimens of 400 mcg vaginally and orally were most commonly practiced in our institution.

Note that the regimens in other groups were varied from intravaginal 25 mcg to oral 100 mcg, which seem to be too low for the second-trimester termination of pregnancy. The use of low dose regimens was likely associated with concerns about misoprostol-induced uterine hyperstimulation in cases of an advanced gestational age, since we realize that the higher the gestational age is, the lower the misoprostol dose needs to be, due to a higher response of the uterus.

The weaknesses of this study include: (1) there was no randomization allocated to the regimen group; (2) the long time frame (3) the retrospective nature of the study; (4) the various regimens of misoprostol, especially in the groups of other regimens; (5) and there were no comparisons of misoprostol use with other methods, such as other types of prostaglandins or high-dose oxytocin. However, a previous report demonstrated a higher efficacy of misoprostol compared to high-dose oxytocin [7]. Finally, our results should be interpreted with caution since multivariate analysis to control several confounding factors, such as racial factors, was not performed. Accordingly, a universal reproducibility in clinical practice may not be perfectly reproduced among other populations.

The strengths of this study are as follows: (1) the large sample size, especially the top three most common regimens—to the best of our knowledge, this is the largest series of TOP of DFIU; (2) the homogeneous population, specific to the group of DFIU with no cases of live fetuses confined in the second trimester; (3) the homogeneous use of medication, including only misoprostol though several regimens; (4) the prospective data collection; and (5) the findings representing real practice rather than ideal conditions of a research setting, thereby enabling our findings’ reproducibility in clinical use may be expected.

## 5. Conclusions

This study gives promising evidence of the high effectiveness and high safety of misoprostol in the termination of a DIFU in the second trimester. In the aspect of global health, either 400-mcg misoprostol administered intravaginally every 6 h or orally every 4 h or through the combination route can be a good option in clinical practice, especially in geographical areas with low-resource levels.

## Data Availability

The datasets analyzed during the current study are available from the corresponding author upon reasonable request.

## References

[1] Liu L.C., Huang H.B., Yu M.H., Su H.Y. (2013). Analysis of intrauterine fetal demise—A hospital-based study in Taiwan over a decade. Taiwan J. Obstet. Gynecol..

[2] Man J., Hutchinson J.C., Ashworth M., Heazell A.E., Jeffrey I., Sebire N.J. (2016). Stillbirth and intrauterine fetal death: Contemporary demographic features of >1000 cases from an urban population. Ultrasound Obstet. Gynecol..

[3] Beucher G., Dolley P., Stewart Z., Carles G., Grossetti E., Dreyfus M. (2015). Fetal death beyond 14 weeks of gestation: Induction of labor and obtaining of uterine vacuity. Gynecol. Obstet. Fertil..

[4] Clouqueur E., Coulon C., Vaast P., Chauvet A., Deruelle P., Subtil D., Houfflin-Debarge V. (2014). Use of misoprostol for induction of labor in case of fetal death or termination of pregnancy during second or third trimester of pregnancy: Efficiency, dosage, route of administration, side effects, use in case of uterine scar. J. Gynecol. Obstet. Biol. Reprod..

[5] Pongsatha S., Tongsong T. (2014). Randomized controlled trial comparing efficacy between a vaginal misoprostol loading and non-loading dose regimen for second-trimester pregnancy termination. J. Obstet. Gynaecol. Res..

[6] Pongsatha S., Tongsong T. (2004). Therapeutic termination of second trimester pregnancies with intrauterine fetal death with 400 micrograms of oral misoprostol. J. Obstet. Gynaecol. Res..

[7] Abediasl Z., Sheikh M., Pooransari P., Farahani Z., Kalani F. (2016). Vaginal misoprostol versus intravenous oxytocin for the management of second-trimester pregnancies with intrauterine fetal death: A randomized clinical trial. J. Obstet. Gynaecol. Res..

[8] Nakintu N. (2001). A comparative study of vaginal misoprostol and intravenous oxytocin for induction of labour in women with intra uterine fetal death in Mulago Hospital, Uganda. Afr. Health Sci..

[9] de León R.G.P., Wing D.A. (2009). Misoprostol for termination of pregnancy with intrauterine fetal demise in the second and third trimester of pregnancy—A systematic review. Contraception.

[10] Ngoc N.T.N., Shochet T., Raghavan S., Blum J., Nga N.T.B., Minh N.T.H., Phan V.Q., Winikoff B. (2011). Mifepristone and misoprostol compared with misoprostol alone for second-trimester abortion: A randomized controlled trial. Obstet. Gynecol..

[11] Rose S.B., Shand C., Simmons A. (2006). Mifepristone- and misoprostol-induced mid-trimester termination of pregnancy: A review of 272 cases. Aust. N. Z. J. Obstet. Gynaecol..

[12] Maria B., Matheron I. (1994). Methods of second trimester pregnancy termination and evacuation of in utero dead fetuses. Value of mifepristone. J. Gynecol. Obstet. Biol. Reprod..

[13] Srisomboon J., Pongpisuttinun S. (1998). Efficacy of intracervicovaginal misoprostol in second-trimester pregnancy termination: A comparison between live and dead fetuses. J. Obstet. Gynaecol. Res..

[14] Lemmers M., Verschoor M.A., Kim B.V., Hickey M., Vazquez J.C., Mol B.W.J., Neilson J.P. (2019). Medical treatment for early fetal death (less than 24 weeks). Cochrane Database Syst. Rev..

[15] Pongsatha S., Tongsong T. (2004). Intravaginal misoprostol for pregnancy termination. Int. J. Gynaecol. Obstet..

